# Cytomegalovirus-induced immunopathology and its clinical consequences

**DOI:** 10.1186/2042-4280-2-6

**Published:** 2011-04-07

**Authors:** Stefania Varani, Maria Paola Landini

**Affiliations:** 1 Section of Microbiology, Department of Hematology and Oncology, University of Bologna, Bologna, Italy

## Abstract

Human cytomegalovirus (CMV) is a ubiquitous DNA virus that causes severe disease in patients with immature or impaired immune systems. During active infection, CMV modulates host immunity, and CMV-infected patients often develop signs of immune dysfunction, such as immunosuppression and autoimmune phenomena. Furthermore, active viral infection has been observed in several autoimmune diseases, and case reports have linked primary CMV infection and the onset of autoimmune disorders. In addition, CMV infection promotes allograft rejection and graft-versus-host disease in solid organ and bone marrow transplant recipients, respectively, further implicating CMV in the genesis and maintenance of immunopathological phenomena. The mechanisms by which CMV could induce inhibition of host defense, inflammation, and autoimmunity are discussed, as is the treatment of virus-induced immunopathology with antivirals.

## Human cytomegalovirus

Human cytomegalovirus (CMV) is a widespread agent that belongs to the *Herpesviridae *family [1]. Viral proteins are expressed in the immediate early (IE), early (E), and late (L) phases of CMV infection. Its genome contains more than 200 potential reading frames from which effector proteins can be generated, but merely one-quarter is committed to replication [2,3]. Thus, the majority of viral proteins potentially modulates cellular responses in the host; of all herpesviruses, CMV expresses the most genes that alter innate and adaptive host immune responses [4].

During the acute phase of CMV infection, many cell types in an organ system can be infected, including endothelial cells, epithelial cells, smooth muscle cells, fibroblasts, neuronal cells, hepatocytes, trophoblasts, monocytes/macrophages (Mϕs), and dendritic cells (DCs) [5]. The virus typically is acquired early in life and can be transmitted by direct or indirect contact with infected body fluids. There are 3 forms of active CMV infection: a) primary infection, which occurs when the virus infects a CMV-naive host; b) endogenous infection in CMV-seropositive individuals who experience reactivation from latency, and c) exogenous reinfection in previously infected individuals who experience infection by a different strain [6].

Recent evidence shows that active and latent CMV infection induces sustained systemic inflammatory responses that are accompanied by a type 1 cytokine signature [7]. Viral persistence is established in all infected individuals and is chronically productive or occurs as a latent infection in which viral gene expression is limited [8].

Initiation of viral replication from latency not only is caused by immunosuppression but, like other viruses, such as HIV [9], also appears to be linked to activation of the immune system. For example, the virus can be reactivated by tumor necrosis factor (TNF)- α, which is released during inflammation. TNF-α binds to the TNF receptor on latently infected cells, generating signals that activate nuclear factor-kB (NF-kB). Consequently, the activated p65/p50 NF-kB heterodimer translocates into the nucleus and binds to the IE enhancer region of CMV, which initiates viral replication [10].

This molecular mechanism has a clinical correlate, wherein the reactivation of latent CMV has been associated with elevated serum levels of TNF-α in patients with atopic dermatitis [11] and sepsis [10,12,13]. In addition, CMV reactivates commonly following acute rejection of organ transplants and after acute graft-versus-host disease (GVHD) in bone marrow transplant (BMT) recipients who have elevated TNF-α levels [14-17].

Further, proinflammatory prostaglandins stimulate cyclic AMP, which then triggers viral reactivation [18]. Stress catecholamines can induce increases in cyclic AMP concentrations, leading to viral reactivation [6,19]. Through such mechanisms, chronic inflammation is likely to mediate the reactivation of CMV.

Cells of the myeloid lineage are carriers of latent CMV [20,21]. CMV can reactivate from latency by allogeneic stimulation of monocytes from seropositive donors [22]. Viral reactivation also occurs when mononuclear hematopoietic progenitors that are latently infected with CMV differentiate into mature DCs [23]. Thus, inflammation and cellular differentiation are events that reactivate CMV.

## Clinical features of CMV infection and disease

### CMV infection in immunocompetent hosts

In adults, primary CMV infection occurs in 0.1% to 0.6% of blood donors and typically is prolonged [24,25]. Immunocompetent individuals with primary infections are frequently asymptomatic [25,26], but CMV occasionally effects clinical illness-i.e., a self-limited mononucleosis-like syndrome. Clinically, the mononucleosis that is caused by CMV is similar to the more common Epstein-Barr virus (EBV) mononucleosis. Malaise, headache, and high fever are hallmarks of CMV mononucleosis and can persist for weeks. Other clinical abnormalities have been associated with CMV infection in normal hosts, including Guillain-Barré syndrome, meningoencephalitis, hemolytic anemia, and thrombocytopenia [1].

### CMV infection in immunocompromised patients

CMV infections are among the most common infections that follow transplantation. In such transplant recipients, CMV infection manifests as a wide range of conditions, from asymptomatic viremia to CMV syndrome and tissue-invasive disease [27].

CMV infection in immunocompromised individuals causes disparate clinical syndromes in different groups of patients, and the severity of infection is proportional to the degree of immunosuppression. The most severe infections develop in allogeneic bone marrow and allogeneic stem cell transplant (alloSCT) recipients and in AIDS patients with low CD4^+ ^counts. Symptomatic CMV infections are also observed often in solid organ transplant recipients.

The effects of CMV infection in transplant patients can be divided into 2 categories: direct effects of the infection that cause mononucleosis-like syndrome or tissue-invasive disease, and indirect effects [28-30]. CMV tissue-invasive disease is suspected if high levels of CMV viremia develop and is confirmed by detection of the virus in the affected tissue by immunohistochemistry. The transplanted organ is the principal target of CMV infection in solid organ recipients [30]. This is not the case in BMT recipients, where CMV disease frequently manifests as interstitial pneumonia [31].

CMV is also associated with indirect effects, a term that encompasses the effects that are coupled to longer periods of low viral replication and that are caused in part by the host's immune response. Such effects include graft rejection and immunosuppression.

### CMV infection in patients with autoimmune disorders

Recently, laboratory-based signs of active CMV infection have been observed in association with the onset and course of autoimmune diseases, as reviewed extensively below.

## The virus as an immunopathological agent: autoimmunity, immunosuppression, and graft rejection

### A. Autoimmunity

#### A1. Induction of autoantibodies

Autoimmune phenomena often develop in CMV-infected patients. For example, anti-phospholipid and anti-CD13 autoantibodies have been observed in CMV-infected BMT recipients [32-34], and anti-CD13 has been linked to the development of chronic GVHD in these patients [35]. In solid organ transplant recipients, non-organ-specific autoantibodies, such as anti-endothelial cell, anti-smooth muscle cell, and anti-nucleus antibodies, are associated with CMV infection [36,37], likely increasing the risk for humoral and chronic allograft rejection [38,39]. In addition, hypergammaglobulinemia, cryoglobulinemia, and autoantibody production are features of CMV-induced mononucleosis and postperfusion syndrome [40-42].

#### A2. Induction of vasculitides and scleroderma

The time course of active CMV infection and the onset of autoimmune disorders have been linked in previously healthy individuals. Notably, the presence of CMV replication has been associated with the development of autoimmune vasculitis and scleroderma, implicating virus-induced vasculopathy as a trigger of autoimmunity.

In fact, active CMV infection has been correlated with newly diagnosed necrotizing vasculitis [43], cutaneous vasculitis [44], and systemic lupus erythematosus (SLE)-associated vasculitis [45]. In all but one case, the symptoms improved with ganciclovir, in association or not with prednisone.

Further, a previously healthy woman who acquired CMV mononucleosis developed vasculitis with antineutrophil cytoplasmic antibodies (c-ANCA). This patient had extraordinarily high plasma levels of IL-5 and lymphotoxin-α and developed autoantibodies, concomitant with the primary CMV infection. After the onset of vasculitis, CMV genomes were detected in the blood and urine, and CMV antigens were observed in inflammatory lesions of the kidney, suggesting that CMV triggers and maintains the autoimmune process [42].

Finally, CMV RNA was detected in endothelial cells from skin biopsies in patients who presented with sudden onset of autoimmune sclerosis. One patient was treated with ganciclovir, which did not improve the clinical course of the disease [44].

#### A3. Induction of encephalitis associated with autoimmune phenomena

Recently, a previously healthy woman who suffered from active CMV experienced abrupt onset of encephalitis that was associated with autoimmune phenomena. Primary CMV infection was diagnosed by serology, and CMV DNA was detected in the cerebrospinal fluid and blood at the onset of symptoms. Long-course treatment with ganciclovir and intravenous immunoglobulins and decreasing doses of cortisone improved the neurological status. Although viral replication was halted and immunosuppressive therapy was discontinued, extremely low levels of CMV-specific CD4^+ ^and CD8^+ ^T cells were detected for up to 10 months after disease onset. Conversely, high blood interferon (IFN)- γ levels were observed, suggesting enhancement of nonspecific immune mechanisms that were activated to compensate for the lack of CMV-specific T cell responses (Xu, Varani et al., manuscript in preparation). This case suggests that CMV infection in subjects with potentially hidden immune defects can enhance viral replication that triggers autoimmune phenomena.

Although it is possible that they are unrelated with regard to causation, the concurrent active CMV infection and onset of autoimmunity that were observed in these cases suggest that CMV induces autoimmunity in predisposed individuals.

#### A4. Increased risk for post-transplant diabetes mellitus

Viral infections, such as enteroviruses and mumps virus, are believed to provoke type I diabetes in genetically predisposed individuals [46]. Clinical evidence suggests that asymptomatic CMV infection and CMV disease are independent risk factors for early-onset diabetes mellitus in recipients of renal transplant (generally referred to as PTDM) [47,48]. Further, CMV donor-positive/recipient-negative serostatus is a risk factor for the development of PTDM in pediatric renal transplant patients [49], and active CMV infection predisposes adult liver transplant patients to the development of PTDM [50]. The incidence of PTDM has declined significantly since the introduction of preemptive anti-CMV regimens, supporting the link between CMV and PTDM [51].

CMV damages β-cells by direct viral infection (the pancreas is a target organ of CMV infection [52]), through the cytotoxic effects of activated effector lymphocyte infiltrates, or the induction of proinflammatory cytokines [53]. There are limited experimental data on the inhibition of β-cell function by CMV, however, necessitating additional studies to demonstrate a causal relationship between CMV infection and PTDM.

#### A5. Active infection during autoimmune disorders

Current findings suggest that latent CMV can be reactivated by allogeneic stimulation in monocytes from seropositive donors [22] and that IFN-γ and TNF-α are necessary for the differentiation of CMV-permissive Mϕs [54]. These findings have clinical implications, because immune-mediated processes that involve T cell activation and inflammation may facilitate the reactivation of latent CMV in monocytes *in vivo*. Thus, the chronic inflammation that is associated with autoimmune diseases might provide the ideal microenvironment in which latent CMV can be reactivated in Mϕs; this inflammation can induce DC maturation, which can also provoke viral reactivation from latency [23].

##### Inflammatory bowel diseases and other enteropathies

CMV replicates efficiently in epithelial cells of the intestinal mucosa [55,56] and is a major cause of graft failure after intestinal transplantation [57]. In recent years, many studies have focused on the pathogenic function of CMV replication in inflammatory bowel disease (IBD). Notably, CMV antigens have been found in 10% to 90% of biopsies from patients with IBD [58-60]. Patients with inactive or mild to moderate ulcerative colitis (UC) [60,61] and Crohn disease [60-63] rarely show signs of CMV replication, whereas active CMV infection exists in 20% to 40% of steroid-refractory UC [63-73], suggesting that CMV exacerbates inflammation.

Antiviral treatment in patients with steroid-resistant UC and active CMV infection has been efficacious in isolated cases or small groups of patients [63,65,68,70,71,74], whereas other studies have reported clinical improvements in CMV colitis in the absence of antiviral medication [60,75,76], indicating the need for large, randomized, controlled studies to determine the true clinical value of antivirals in CMV-positive UC.

Notably, active CMV infection was diagnosed based on the detection of CMV in mucosal biopsy specimens from the colon by immunohistochemistry and PCR in the majority of these studies [60,63-66,68-70,72,77], whereas blood analysis, when performed, demonstrated low viral loads or the absence of viremia [63,64]. These findings imply that CMV replication occurs primarily in the colon of patients with UC.

Further, recent evidence has shown that all patients with steroid-refractory UC who have experienced active CMV infection have been previously CMV-seropositive, suggesting that the virus reactivated at the site of inflammation during the active phase of disease [63]. Latently infected monocytes [20] are recruited to the site of colonic inflammation, where monocyte activation and differentiation can induce viral reactivation [22,23]. Whether epithelial cells of the colonic mucosa carry latent CMV that contributes to viral reactivation is unknown.

Evidence indicates that CMV DNA can also be detected in intestinal biopsies of patients with common variable immunodeficiency (CVID) [78]. CVID is a heterogeneous disease that results in hypogammaglobulinemia, a propensity for infection and autoimmunity and that may also complicate with severe enteropathy. The role of CMV infection in triggering/worsening such disease is only partially understood; preliminary clinical observations suggest that an exaggerated T cell response to CMV may cause or exacerbate enteropathy in CVID [79], further underscoring the potential inflammatory role of CMV in the gastrointestinal tract.

##### Autoimmune disorders with major vascular involvement: vasculitis and systemic sclerosis

Increasing evidence suggests that in addition to hepatitis C (HCV), other viruses, such as CMV, EBV, HIV, and parvovirus B19, accompany systemic vasculitis [80]. In such disorders, CMV infection coincides with the onset of inflammatory disease [42-45], as discussed, or with the initiation of immunosuppressive therapy [81]. Recently, IgM antibodies against CMV were observed more frequently in patients with c-ANCA-positive vasculitis compared with controls, while no other viral, bacterial and parasitic infections appeared to be involved [82]. Thus, CMV infection might initiate or maintain inflammation in vasculitides.

CMV has also been implicated as a trigger of vascular damage in systemic sclerosis [83]. The clinical onset of systemic sclerosis has been associated with the presence of an active CMV infection [44]. Further, autoantibodies that are specific for systemic sclerosis recognize the late CMV protein UL94 and are associated with the diffuse form of the disease but not the limited form, suggesting a correlation between the virus and disease severity [84,85]. Notably, antibodies against UL94 induce apoptosis in endothelial cells and activate dermal fibroblasts *in vitro*, effecting 2 hallmarks of systemic sclerosis-vascular damage and fibrosis [86].

##### Other autoimmune disorders

Laboratory signs of acute CMV infection and anti-CMV have been observed in other autoimmune diseases. Subclinical systemic CMV infection develops in psoriatic patients and is associated with high levels of TNF-α [87]. Moreover, CMV DNA, specific antigens, and infectious virus particles have been detected in synovial tissue and fluid from the joints of 10% to 50% patients with rheumatoid arthritis (RA) [88-91].

Active CMV infection is also frequent in children with SLE [92], and CMV has been implicated in its development and exacerbation [93-97]. Serological signs of active CMV infection have been detected in 10% of patients with SLE, and the presence of viral infection is associated with higher disease activity scores [98]. Patients with SLE also have more robust humoral activity in response to CMV [99] and, in particular, to the CMV structural protein pp65 [100] compared with healthy donors and patients with other autoimmune disorders. In a recent study, CMV was the only infectious agent for which higher rates of IgM seropositivity and higher antibody titers were observed in SLE patients versus controls [101].

### B. Immunosuppression

CMV infection (mainly primary infection) causes transient but substantial immunosuppression [102]. CMV effects immunosuppression in solid organ transplant recipients, potentiating superinfections with various pathogens [103]. Notably, meta-analyses of thousands of transplant recipients have demonstrated significant effects of anti-CMV prophylaxis in preventing bacterial and fungal [104] infections and bacterial and protozoan [105] infections. Further, in solid organ recipients, CMV replication influences the viral load of other viruses, such as human herpes virus (HHV)-6 and HHV-7 [106], and HCV load [107].

Owing to its immunosuppressive effect, CMV has also been suggested as a risk factor for the development of post-transplant lymphoproliferative disorders (PTLDs) in solid organ recipients, a pathological condition that is associated strictly with EBV replication [108]. In particular, CMV mismatch (donor positive/recipient negative) [109] and CMV disease [110] have been identified as general risk factors predisposing to the development of PTLD in solid organ transplant recipients. However, recent evidence shows no correlation between CMV disease and the development of PTLD [111-113]. Therefore, whether CMV is associated to an increased risk of PTLD is debatable and further studies are needed to clarify this matter.

CMV is immunosuppressive in allo-SCT recipients and death appears to be mediated by invasive bacterial and fungal infections, of which invasive aspergillosis is the most significant complication [114]. In addition, by preventing CMV replication in BMT recipients, a reduction in mortality as a result of all types of infections is achieved [115]. Finally, an increased risk of death from infections has been shown in CMV-seropositive BMT recipients receiving grafts from seronegative donors [114,116,117], supporting the importance of transferring specific T cells with the graft to control CMV replication and its immunomodulatory effects in this patient's cohort.

### C. Graft rejection

CMV promotes classical rejection and vasculopathy of an allograft, which impacts its longevity [6]. Several cohort studies have shown that CMV infection is associated with an increased risk of graft rejection in renal, liver, and lung transplant patients [118-121].

Studies in heart transplant recipients report that acute rejection and accelerated coronary atherosclerosis are linked to asymptomatic and symptomatic CMV infection [122,123]. Cardiac transplant vascular sclerosis, characterized histologically by diffuse concentric intimal proliferation that results in vessel stenosis and, ultimately, allograft failure [124], is highly associated with CMV in heart transplant recipients [125]. The higher incidence of viral DNA in explant vascular intima from patients with cardiac graft vascular sclerosis compared with explants without vasculopathy underscores the influence of CMV on the development of chronic rejection [126]. In addition, the early control of subclinical replication of CMV after cardiac transplantation by T cell immunity may reduce allograft vasculopathy and allograft rejection [127].

Chronic vasculopathy has also been associated with CMV in kidney transplant recipients [128]; bronchiolitis obliterans in lung recipients also correlates with CMV infection [119,129]. Several randomized trials of antiviral prophylaxis and preemptive therapy have demonstrated that antivirals provide significant protection against CMV-associated allograft injury, providing the strongest evidence for the link between CMV infection and allograft rejection [104,130-133].

In BMT recipients, GVHD and CMV replication are pathogenetically associated; multiple studies show that GVHD and its treatment put patients at risk for CMV replication [134-136]. In contrast, the role of CMV replication as a cause of GVHD is controversial. Opposite findings have been published on the effect of CMV replication on development of acute GVHD [116,136-139]. Several studies showed that an increased risk of chronic GVHD was associated with CMV viremia [35,137,140] and that BMT patients receiving pre-emptive therapy for CMV replication exhibited lower risks for severe chronic GVHD [141]. On the other hand, large randomized studies of prophylaxis with acyclovir or valacyclovir showing effects on CMV replication did not have any impact on the risk for GVHD [115,142].

## Mechanisms of CMV-induced immunopathology

### A. Humoral autoimmunity

**Figure 1:**
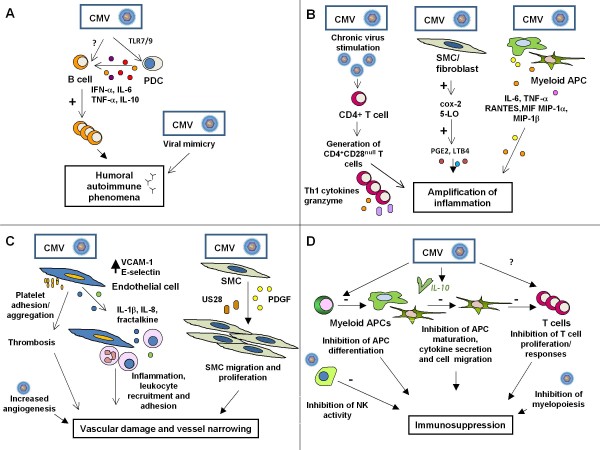
**Mechanisms by which CMV can induce host immunopathology**. A; CMV-induced autoantibody production. B; Enhanced inflammation caused by the virus. C; CMV-induced vascular damage and vessel thickening. D; CMV-induced immunosuppression. TLR7/9; toll-like receptor 7/9, PDC; plasmacytoid dendritic cell, SMC; smooth muscle cell, 5-LO; 5-lypooxygenase, cox-2, cyclooxygenase-2, PGE2; prostaglandin E2, LTB4; leukotriene B4, MIF; macrophage migration inhibitory factor, MIP-1α; macrophage inflammatory protein 1-α, MIP-1β; macrophage inflammatory protein 1-β, VCAM-1; vascular cell adhesion molecule-1, PDGF; platelet-derived-growth factor, vIL-10; virally encoded IL-10. Modified from: Varani et al. "Cytomegalovirus-induced autoimmunity" in "Autoimmune Disorders: Symptoms, Diagnosis and Treatment". Editor: M.E. Petrov. ISBN: 978-1-61761-552-8; ^© ^2010 Nova Science Publishers, Inc.

The mechanisms by which CMV interacts with the immune response to induce autoimmune phenomena are unknown. One possibility is viral mimicry [143]. The CMV genome harbors a series of genes that are homologous to cellular genes; consequently, the host response to viral determinants can crossreact with host tissues, leading to autoimmunity (Figure 1A). This mechanism likely explains the generation of pathogenetic autoantibodies that crossreact with CMV during systemic sclerosis [83].

Humoral autoimmunity can also be elicited by nonspecific B cell activation that is caused by CMV. CMV is a polyclonal B-cell activator *in vitro*, and the B cell hyperresponse does not require viral replication [144]. In addition, CMV interacts with toll-like receptor (TLR) 7 and/or 9 in human plasmacytoid DCs, leading to secretion of IFN-α and B cell proliferation [145]. These DC-mediated events might facilitate polyclonal B cell activation and autoantibody production during CMV infection (Figure 1A).

B cell hyperactivation has clinical implications for infected patients, as demonstrated in transplant recipients, wherein autoantibodies contribute to the development of GVHD in CMV-infected alloSCT patients and to graft rejection in solid organ recipients [34,35,38,39].

### B. Inflammation

Primary and latent CMV infections induce chronic, systemic type 1 inflammatory responses [7]. Such sustained immune activation can augment alloimmune responses by enhancing the expansion and function of alloreactive T cells after transplantation. Moreover, the protracted elevation in serum IFN-γ levels can increase major histocompatibility complex (MHC) expression on graft cells, raising the risk of recognition by alloantigen-specific T cells. Both mechanisms can mediate allograft rejection.

The primary involvement of IFN-γ, T-bet (functional markers of Th1 CD4^+ ^T cells), and granzyme B (a cytotoxic marker of CD8^+ ^cells) in the pathogenesis of transplant glomerulopathy [146], a major risk factor for chronic graft rejection, has been demonstrated recently, implicating Th1-inducing CMV in chronic rejection in renal transplant recipients. Immune-mediated damage, involving CD8^+ ^granzyme^+ ^cytotoxic T cells, has also been observed in fetuses that are severely affected by congenital CMV infections (Gabrielli, Landini et al., manuscript in preparation). As discussed below, inflammation might enhance autoimmunity when CMV has reactivated in autoimmune diseases.

#### Generation of CD4^+ ^CD28^null ^T cells

A unique subset of CD4^+ ^T cells that lack the costimulatory molecule CD28 expand in patients with autoimmune diseases, such as RA, Wegener's granulomatosis, dermatomyositis and polymyositis, multiple sclerosis, and IBD [147-150]. These cells have pathogenic properties *in vitro *[151], are a major source of Th1 cytokines in lesions in Wegener's granulomatosis [152], and are associated with early atherosclerotic vessel damage in RA patients [153]. In addition, CD4^+^CD28^- ^and CD8^+^CD28^- ^T cells are the predominant T cells that infiltrate inflamed muscles in patients with dermatomyositis and polymyositis, secreting IFN-γ on CMV-specific antigenic stimulation [150].

Notably, CD4^+^CD28^- ^T cells appear to exist almost exclusively in CMV-infected individuals [150,154]. In RA patients and healthy controls, CD4^+^CD28^- ^lymphocytes react specifically with several CMV epitopes [151]. Thus, CMV replication in inflammatory lesions has been speculated to drive the differentiation of CD4^+ ^T cells into pathogenic CD28^null ^T cells, thereby aggravating local chronic inflammation in autoimmune disorders [151] (Figure 1B).

#### NF-kB and other inflammatory factors

In addition to inducing the end-stage differentiation of pathogenic T cells, CMV sustains chronic inflammation through other mechanisms. *In vitro*, CMV infection stimulates the translocation of NF-kB into the nucleus, which then upregulates TNF-α, leading to further activation of latent CMV and inflammatory responses [155].

CMV induces transient cyclooxygenase 2 expression in infected fibroblasts and the subsequent release of prostaglandin E2, a mediator of inflammation [156]. CMV also stimulates 5-lypoxygenase expression, which is crucial for the synthesis of leukotriene B4, a powerful chemoattractant [157] (Figure 1B). In addition, CMV infects various subsets of myeloid antigen-presenting cells (APCs) efficiently [158-160] that, once infected, release myriad inflammatory mediators [160-162] (Figure 1B). These mechanisms might sustain inflammation in CMV-infected lesions in autoimmune disorders and CMV-positive allografts.

### C. Vascular damage and stenosis

Viral infections mediate the pathogenesis of vascular damage and vascular stenosis through various mechanisms, such as the infection of endothelial cells, causing cellular dysfunction or death; immune-mediated injury of the vessel wall; hemorheological dysfunction due to increased procoagulant activity; and migration and proliferation of smooth muscle cells [163-165].

CMV can productively infect endothelial cells *in vitro *[166-168] and CMV-infected endothelial cells are dysfunctional, due to diminished expression and activity of endothelial nitric oxide synthase [169]; augmented release of IL-8, a regulator of neutrophil migration [170]; increased secretion of the proinflammatory cytokine IL-1β; and upregulation of adhesion molecules that promote leukocyte adhesion [171] (Figure 1C).

The function of virus-induced cytokines and chemokines in the initiation and exacerbation of vascular damage is a growing area of research. CMV induces the release of proinflammatory cytokines and chemokines and encodes CC and CXC chemokine homologs that recruit cellular infiltrates [165]. In addition, during host CD4^+ ^T cell responses to CMV antigens, sufficient levels of IFN-γ and TNF-α are generated to induce the expression of fractalkine in endothelial cells [172]. On such upregulation, fractalkine mediates the recruitment and mobilization of natural killer (NK) cells and monocytes, which damage endothelial cells [173]. Thus, CMV-associated chronic endothelial cell inflammation and damage result from chemokine-mediated immunopathogenic effects (Figure 1C).

CMV infection can modulate the activity of the endothelium-from anticoagulant to procoagulant [174]-and induce platelet adherence and aggregation in infected endothelium [175]. Such effects can aggravate the vascular damage that is induced by CMV and induce vascular inflammation (Figure 1C). Endothelial cell damage, cytokine and chemokine release, and cellular dysfunction likely contribute to allograft-associated vasculopathy [165].

In addition to endothelial cells, CMV productively infects all cell types that are involved in vascular rejection, including smooth muscle cells (SMCs), Mϕs, and fibroblasts. The migration of SMCs from the media into the neointimal space and their subsequent proliferation are hallmarks of the development of vascular lesions during allograft vasculopathy.

CMV blocks apoptosis through various mechanisms [176], which effects the accumulation of SMCs. CMV also induces the production of potent stimuli of SMC proliferation, such as platelet-derived growth factor [177]. In addition, by generating the chemokine receptor US28, CMV enhances SMC migration [178]. The resulting accumulation of SMCs in the vessel intima on CMV infection leads to neointimal hyperplasia and vessel narrowing. Activated inflammatory cells, fibroblasts, and SMCs within vascular lesions are important local sources of factors that promote angiogenesis [179], which accelerates the development of transplant vascular sclerosis (Figure 1C).

### D. Immunosuppression

Because CMV induces a robust and chronic antigenic response in immunocompetent individuals that increases with age [180], it has evolved several mechanisms to suppress and evade this response and persist in the host. Such mechanisms lead to transient but substantial immunosuppression against the virus itself and unrelated pathogens [181].

CMV-induced impairments that impede host immune responses have been demonstrated *in vivo *and *in vitro*. For example, patients with CMV mononucleosis experience a loss in delayed-type hypersensitivity reactions to recall antigens [182] and reduced lymphoproliferative responses to mitogens [183] and specific antigens [184]. In immunocompetent adolescents with asymptomatic primary CMV infections, lymphocyte proliferation in response to CMV is less robust than in seropositive controls [26]. Specific cell-mediated immunity is also attenuated in children with congenital [185] and acquired [186] CMV infections.

*In vitro*, CMV suppresses lymphocyte proliferation to T cell mitogens and prevents lymphocytes and monocytes from producing and responding to immune mediators, such as IL-1 and IL-2 [187]. In addition, CMV inhibits cytotoxic and NK cell activity [188,189]. CMV suppresses bone marrow myelopoiesis by infecting hematopoietic progenitors and their progeny directly or infecting stromal cells and altering the bone marrow microenvironment [190-192].

These observations are supported by evidence of impaired APC function on infection with CMV, including altered phagocytosis, differentiation, migration and maturation, and reduced expression of MHC molecules, preventing effective antigen presentation to T cells [160,162,187,193-199]. CMV impairs the ability of plasmacytoid DCs to induce allogeneic T cell proliferation [145], indicating broad virus-induced inhibition of various APC subsets.

The secretion of cmvIL-10 during CMV infection might aid the virus in infecting and inhibiting DCs chronically [200,201] (Figure 1D). CMV might use these mechanisms as immunoevasive strategies and simultaneously effect robust and broad inhibition of host immunity.

## Treatment of virus-induced immunopathology

Despite the substantial progress in transplantology, CMV continues to be a significant cause of morbidity in transplant recipients, due to its many direct and indirect effects. While the direct effects of CMV infection are well managed by treatment with ganciclovir or its prodrug, valganciclovir, the optimal therapy for treating and preventing virus-induced immunopathology remains undefined [6]. Studies indicate that antiviral prophylaxis provides significant protection against CMV-associated allograft injury and immunosuppression [104,105,130,131]. However, no guidelines on the treatment of virus-induced immunopathology in transplant recipients exist. Similarly, little is known about the treatment of viral immunomodulation in patients with autoimmune disorders.

When CMV replication is detected in patients with autoimmune diseases, clinicians are faced with a therapeutic dilemma: should antiviral therapy be initiated and immunosuppression be reduced to generate specific antiviral immune responses, despite the risk of exacerbating the autoimmune disorder? Or, should the doses of immunosuppressive agents be increased to suppress inflammatory activity? Similarly, the treatment of CMV infection that accompanies allograft rejection in solid organ transplant recipients is complicated, because the modulation of iatrogenic immunosuppression can oppositely influence CMV replication and the rejection episode [202].

As discussed, in 5 patients with vasculitis who tested positive for active CMV infection, remission of the autoimmunity was achieved on treatment with ganciclovir alone or with CMV immunoglobulin and/or cortisone [43-45]. We also effected a successful outcome in a patient who developed encephalitis and autoimmune phenomena on primary CMV infection after a long-course treatment with ganciclovir and intravenous immunoglobulins and decreasing doses of prednisone (Xu, Varani et al., manuscript in preparation). The positive outcomes in these cases suggest that a 2-pronged approach-comprising the inhibition of viral replication by antivirals and immunomodulation by intravenous IgG [203] and/or prednisone-is warranted when the onset of autoimmune disorders coincides with active CMV infection.

Increasing evidence shows that CMV exacerbates the clinical outcome of UC, prompting the hypothesis that antiviral therapy or another regimen that impedes viral replication is beneficial when CMV infection is histologically proven at the site of inflammation [63]. To this end, 3 therapeutic options have been considered; 1. administration of antiviral compounds, 2. modulation of immunosuppression, and 3. modulation of inflammation.

Many studies have reported successful outcomes using antivirals, such as ganciclovir and oral valganciclovir, in isolated cases or small groups of patients with steroid-refractory UC and active CMV infection [63,65,68,70,71,74]. Recently, it has been suggested that all UC patients with severe colitis that is refractory to immunosuppressants be tested for CMV reactivation and receive antiviral therapy if colonic CMV is detected [204].

Modulating immunosuppressive therapy elicits stronger anti-CMV immune responses; this option has been used successfully alone [64] or with antivirals [64,67,71].

Because CMV reactivation depends strictly on inflammation [10], treatments that reduce colonic inflammation, such as anti-TNF-α compounds and leukapheresis, can reduce viral replication in UC patients, as shown by 2 recent reports [74,205].

Thus, the use of antivirals and indirect suppression of viral replication might be effective treatments for CMV-positive refractory UC. However, large, randomized, controlled studies are needed to determine their efficacies in UC patients and other patients with autoimmune disorders who experience active CMV infection.

## Conclusion

**Figure 2:**
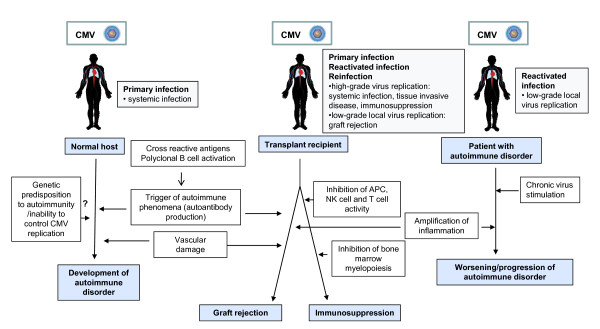
**CMV-induced immunopathology in various groups of patients--previously healthy subjects, immunodepressed transplant recipients, and patients with autoimmune disorders**. Modified from: Varani et al. "Cytomegalovirus-induced autoimmunity" in "Autoimmune Disorders: Symptoms, Diagnosis and Treatment". Editor: M.E. Petrov. ISBN: 978-1-61761-552-8; ^© ^2010 Nova Science Publishers, Inc.

During acute CMV infection, patients often suffer from immunological dysfunctions. Autoimmune phenomena are common in CMV-infected patients, and various autoantibodies have been detected in patients with systemic CMV infection [32-34,36,37,42]. Nonspecific hyperactivation of humoral immunity can impede the development of specific B cell responses-a potential mechanism of viral immune evasion. Such a phenomenon has clinical implications for infected patients, as demonstrated in transplant recipients-autoantibodies mediate the development of GVHD in CMV-infected alloSCT patients and graft rejection in solid organ recipients [34,35,38,39] (Figure 2).

In potentially predisposed patients, primary CMV infections can trigger autoimmune disorders, and vasculitides and scleroderma develop concomitantly with or immediately after active CMV infection in previously healthy, immunocompetent subjects [42-45]. In addition to acute systemic CMV infection, low-grade CMV replication appears to be a frequent event in autoimmune disorders [206]. CMV can accelerate the progression of autoimmune disorders by mimicking autoimmune-mediated tissue destruction and aggravating inflammation. Local viral replication is also associated with chronic perivascular inflammation in solid organ transplant recipients (Figure 2). In these patients, CMV persists in the allograft, but few cells are infected directly by CMV. These findings contrast the global effects that CMV has on the acceleration of vascular stenosis and chronic rejection, suggesting that CMV does not promote vascular disease through direct infection of vessels; instead, it likely acts by indirect mechanisms that in part involve the immune system [179].

Paradoxically, CMV infection, principally primary infection, induces transient but significant immunosuppression, which has clinical consequences during active CMV infection in transplant recipients; such patients develop increased risk for opportunistic infections that can be reduced significantly by antiviral prophylaxis [103-105,114] (Figure 2).

Herpesviruses are archetypal persistent infectious agents that, even in individuals with essentially normal immunity, escape occasionally from normal immune control and cause symptomatic disease. Of all herpesviruses, CMV harbors the most genes that are committed to altering innate and adaptive host immunity [4], and a significant fraction of the T cell repertoire in CMV carriers is directed against this virus [1]. Because CMV persists in the host, it can be erroneously implicated in the pathogenesis of various diseases despite its lack of involvement. Nevertheless, examples of immunopathology that are attributed to CMV continue to accumulate, suggesting that this virus has critical immunomodulatory functions.

## Abbreviations

(alloSCT): allogeneic stem cell transplant; (ANCA): antineutrophil cytoplasmic antibody; (APC): antigen presenting cell; (BMT): bone marrow transplant recipient; (CMV): human cytomegalovirus; (CVID): common variable immunodeficiency; (DC): dendritic cell; (E): early; (EBV): Epstein-Barr virus; (GVHD): graft-versus-host disease; (HCV): hepatitis C virus; (HHV): human herpes virus; (IBD): inflammatory bowel disease; (IE): immediate early; (IFN): interferon; (L): late; (MHC): major histocompatibility complex; (Mϕ): macrophage; (NF-kB): nuclear factor-kB; (NK): natural killer; (PTDM): post-transplant diabetes mellitus; (PTLD): post-transplant lymphoproliferative disorder; (RA): rheumatoid arthritis; (SLE): systemic lupus erythematosus; (SMC): smooth muscle cell; (TLR): toll-like receptor; (TNF): tumor necrosis factor; (UC): ulcerative colitis.

## Competing interests

The authors declare that they have no competing interests.

## Authors' contributions

SV and MPL conceived and wrote the manuscript. The authors read and approved the final manuscript.
